# In Situ Transformed CoOOH@Co_3_S_4_ Heterostructured Catalyst for Highly Efficient Catalytic OER Application

**DOI:** 10.3390/nano14211732

**Published:** 2024-10-29

**Authors:** Abu Talha Aqueel Ahmed, Vijaya Gopalan Sree, Abhishek Meena, Akbar I. Inamdar, Hyunsik Im, Sangeun Cho

**Affiliations:** 1 Division of System Semiconductor, Dongguk University, Seoul 04620, Republic of Korea; abutalha.aa@dongguk.edu (A.T.A.A.); abhishek@dongguk.edu (A.M.); akbarphysics@dongguk.edu (A.I.I.); hyunsik7@dongguk.edu (H.I.); 2 Department of Physics, Dongguk University, Seoul 04620, Republic of Korea; sreevg@dongguk.edu

**Keywords:** hydrothermal growth, anion exchange, water electrolysis, heterostructure, oxygen evolution reaction

## Abstract

The deprived electrochemical kinetics of the oxygen evolution reaction (OER) catalyst is the prime bottleneck and remains the major obstacle in the water electrolysis processes. Herein, a facile hydrothermal technique was implemented to form a freestanding polyhedron-like Co_3_O_4_ on the microporous architecture of Ni foam, its reaction kinetics enhanced through sulfide counterpart transformation in the presence of Na_2_S, and their catalytic OER performances comparatively investigated in 1 M KOH medium. The formed Co_3_S_4_ catalyst shows outstanding catalytic OER activity at a current density of 100 mA cm^−2^ by achieving a relatively low overpotential of 292 mV compared to the pure Co_3_O_4_ catalyst and the commercial IrO_2_ catalyst. This enhancement results from the improved active centers and conductivity, which boost the intrinsic reaction kinetics. Further, the optimized Co_3_S_4_ catalyst exhibits admirable prolonged durability up to 72 h at varied current rates with insignificant selectivity decay. The energy dispersive X-ray spectroscopy (EDX) and Raman spectra measured after the prolonged OER stability test reveal a partial transformation of the active catalyst into an oxyhydroxide phase (i.e., CoOOH@Co_3_S_4_), which acts as an active catalyst phase during the electrolysis process.

## 1. Introduction

Hydrogen (H_2_) has emerged as a highly promising, environmentally friendly energy carrier for a sustainable future in the face of growing environmental challenges and energy shortages [1,2,3]. Owing to the enormously high energy density of 142 MJ kg^−1^, H_2_ presents enormous potential as a clean energy source to lessen dependency on fossil fuels, which is ~70% of the total energy needed around the world [4,5,6,7]. The extensive depletion of fossil fuels has led to numerous harmful environmental impacts, including acid rain, air pollution, and global warming. To address these issues, there is an urgent need for an efficient and durable renewable energy system [8]. Utilizing renewable electricity produced by intermittent energy sources like solar and wind power are of great interest, however, their intermittent nature hinders widespread application. Therefore, H_2_ is deemed as a highly efficient and carbon-free energy carrier to fulfill the current energy needs [9,10,11,12]. However, long-term sustainability is impossible with conventional industrial H_2_ production methods like coal gasification and steam methane reforming, which both greatly increase carbon emissions [13,14,15]. In this regard, water electrolysis is becoming more and more popular as a sustainable and ecologically friendly method of producing high-purity hydrogen [16,17,18,19]. The process is restricted despite its potential due to the anode’s slow kinetics of the oxygen evolution reaction (OER), which is mediated by a four-electron transfer mechanism. The overall energy conversion efficiency is hindered by this slow OER, which limits the wider use of water electrolysis for large-scale hydrogen production [20,21,22].

Despite the remarkable OER performance demonstrated by noble metal catalysts like RuO_2_ and IrO_2_, their high cost and constrained availability prevent their widespread industrial use [21,23,24]. As a result, the first row *3d* transition metals (TM) have drawn a lot of attention because of their strong catalytic activity and abundance in the search for more accessible and affordable alternatives [25]. Among all *3d* TM, Co_3_O_4_ have drawn a lot of interest due to their exceptional mechanical, physical, and redox properties, as well as affordability, and therefore, have been studied as non-precious metal catalysts for water electrolysis applications [26,27,28,29,30,31]. However, the scalable utilization is limited due to their poor overpotential (>400 mV at 10 mA cm^−2^), which falls short for their practical utilization at a larger current scale [32]. To address these issues, various synthesis strategies have been implemented including morphology tuning (e.g., nanocubes, spheres, rods, etc.), nano shape or size engineering, and combining or compositing Co_3_O_4_ with other materials (e.g., carbon, graphene, Ag, N, S, etc.) which led to improved material conductivity and enhanced catalytically active sites [33,34]. This is because variation of size or shape from bulk to nano results in enhanced specific surface area, which is in direct relation with the active sites, and the integration of heteroatoms alters the electronic structure of the catalyst materials [17]. The catalytic OER performance of Co_3_O_4_ catalysts can be enhanced by converting oxide into sulfide counter-phase, which can improve both the conductivity and active sites for efficient water electrolysis [35]. It is because the sulfide transformation stands out as a promising approach due to the unoccupied *3d* orbital and the lone pair electrons in the *3p* orbital of sulfur, which are believed to enhance surface charge through donor/acceptor interaction and boost the catalytic OER activity of the Co_3_S_4_ [36,37,38].

Based on the above key consideration, in this work, we report the successful growth of binder-free Co_3_O_4_ with polyhedron-like morphology on the microporous architecture of Ni foam (NF) through a cost-effective hydrothermal process. This formed electrode film then serves as the template to form the desired Co_3_S_4_ via simple anion exchange reactions using the Na_2_S. The catalytic OER activity of the proposed catalyst was enhanced significantly because the substitution of sulfur in the Co_3_O_4_ materials can improve flexibility and promote more efficient electron transport within the structure, which results in enhanced electrochemical performance [39]. The optimized polyhedron-like Co_3_S_4_ catalyst achieves the smaller overpotential (292 mV at 100 mA cm^−2^) and Tafel slope (132 mV dec^−1^) compared to the Co_3_O_4_ (410 mV and 156 mV dec^−1^) catalyst. Further, Co_3_S_4_ sustained the smaller overpotential at a diverse current density range (100 to 500 mA cm^−2^) with a static potential response and sustained prolonged chronopotentiometric stability (72 h) without any significant activity decay. These results position the Co_3_S_4_ as a strong candidate among non-noble metal catalysts for efficient OER in an alkaline medium (Table S1). Besides, the Co_3_S_4_ was converted into the CoOOH@Co_3_S_4_ heterostructure phase upon electrooxidation during the chronopotentiometric test. The viable OER activity is likely attributed to the enhanced electronic conductivity, and the increment in the Co^3+^ active centers upon electrooxidation further contributed to enhancing the efficient electron and ion transport [40].

## 2. Materials and Methods

### 2.1. Materials

All the chemical reagents used in this experiment were of analytical grade and purchased from Sigma-Aldrich, St. Louis, MO, USA. C_2_H_5_NS (≥98%), C_2_H_5_NS (≥98%), KOH (≥85%), CH_3_CH_2_OH (ethanol; ≥95%), HCl (37%), and CH_3_COCH_3_ (≥99.5%) were used as received without any purification. Three-dimensionally (3D) microporous Ni foam with sheet size of 200 mm × 300 mm was purchased from Alantum (Seoul, Republic of Korea).

### 2.2. Synthesis of Co_3_O_4_ and Co_3_S_4_ Electrode Films

The Co_3_O_4_ electrode film was initially deposited on the 3D porous NF (1 × 5 cm^2^) using a hydrothermal process followed by air annealing, and then subsequently, anion exchange procedure was implemented to obtain the desired Co_3_S_4_ electrode film. In a typical synthesis, C_6_H_5_Na_3_O_7_·2H_2_O (6 mmol) and C_2_H_5_NS (6 mmol) were dissolved in 50 mL of deionized water. Thereafter, CoCl_2_·6H_2_O (6 mmol) was added to the mixture under vigorous stirring for about 30 min. The masked NF substrate (1 × 1 cm^2^) and the formed solution were transferred to the Teflon container of the autoclave. The sealed autoclave assembly was kept in a furnace and the deposition was performed at 150 °C for 6 h. The synthesized electrode film was rinsed with deionized water and ethanol and then annealed to get the porous Co_3_O_4_ phase. In the second step, an anion exchange process was performed on the Co_3_O_4_ using Na_2_S (0.1 M) at 120 °C for 10 h to obtain the Co_3_S_4_ electrode film.

### 2.3. Material Characterization

The formed Co_3_O_4_ and Co_3_S_4_ electrode films were characterized by the X-ray diffraction (XRD) measurement technique, which was used to analyze the crystallinity and material structure. The XRD spectra were obtained using a Rigaku Smartlab instrument (Tokyo, Japan) at the spectral angle (2θ) range between 20 and 80° with a scanning speed of 2° min^−1^. The Raman spectroscopy was implemented to investigate the material fingerprints and elemental bonds using a LabRam Aramis instrument (Jobin Yvon, Longjumeau, France). Field emission scanning electron microscope (FE-SEM) coupled with the energy dispersive X-ray spectroscopy (EDX) was employed to evaluate the surface morphology and composition using the JSM-6701F instrument (JEOL, Tokyo, Japan). X-ray photoelectron spectroscopy (XPS) was used to determine the oxidation states of the Co, O, and S constituent elements using a ULVAC PHI 5000 VersaProbe instrument (Kanagawa, Japan), and their respective binding energies were calibrated with the help of carbon (C 1s at 284.30 eV) contaminant as a reference present inside the chamber.

### 2.4. Catalytic OER Testing

A VersaSTAT instrument (Ametek Scientific Instruments, Berwyn, PA, USA) in the standard three-electrode configuration was used to perform all electrochemical OER testing, which includes linear-sweep voltammetry (LSV), non-Faradaic cyclic voltammetry (CV), chronopotentiometry, and electrochemical impedance spectroscopy (EIS) curves. The electrochemical cell was fabricated using a saturated calomel electrode (SCE; reference electrode), graphite rod (counter electrode), synthesized electrode films (working electrode), and 1.0 M KOH solution (electrolyte). To examine the overpotential (*ɳ*) response, LSV curves were recorded in a potential window range between 0.0 and 1.0 V (vs. SCE) and the electrolyte solution was continuously stirred during the OER testing. The obtained potentials were converted into a reversible hydrogen electrode (RHE) reference scale and then *JR* compensation was performed to rectify the ohmic losses caused by internal and electrolyte resistance (*Rs*) as follows [41]:

*E*_RHE_ = *E*_SCE_ + (pH × 0.059) + *E*°_SCE_, (1)

*E*_RHE_ (*JR* compensated) = *E*_RHE_ − (*J* × *Rs*), (2)

*ɳ* = *E*_RHE_ (*JR* compensated) − 1.23, (3)

where *E*_RHE_ represents the converted potential RHE reference scale, *E*_SCE_ stands for the obtained potential in the SCE scale, and *E*°_SCE_ denotes the standard potential of the SCE electrode at room temperature. The non-Faradaic CV curves were recorded in the potential window range between 0.01 and 0.11 V (vs. SCE) as a function of scan rate to estimate the electrochemical double-layer capacitance (*C_DL_*) and electrochemically active surface area (*ECSA*). The electrochemical impedance spectroscopy (EIS) curves were recorded to examine the charge transfer behavior. The EIS curves were measured at a biasing potential of 0.5 V in the broad frequency range between 0.1 and 10 kHz with an AC signal amplitude of 10 mV.

## 3. Results and Discussion

### 3.1. Crystallographic Characteristics

**Figure 1:**
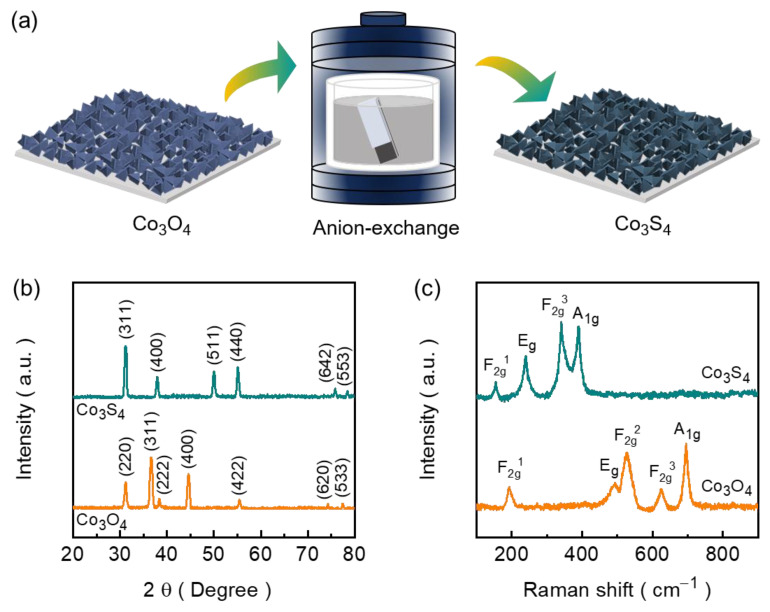
(**a**) Schematic diagram representing the process for the fabrication of Co_3_S_4_ from Co_3_O_4_ through anion exchange procedure. Comparative (**b**) XRD and (**c**) Raman spectra of the prepared Co_3_S_4_ and Co_3_O_4_ electrode films.

Figure 1a shows the schematic representation of the formation of Co_3_S_4_ from Co_3_O_4_ involving an anion exchange reaction, where sulfur anions (S^2−^) replace the oxygen anions (O^2−^) in Co_3_O_4_. The composition and crystalline structure of the films were first determined using the XRD technique. As shown in Figure 1b, the displayed XRD patterns of the synthesized films (Co_3_O_4_ and Co_3_S_4_) exhibit distinct diffraction peaks, indicating both samples possess good crystallinity. The characteristic peaks of Co_3_O_4_ (JCPDS card No. 76-1802) at 31.18, 36.64, 38.30, 44.54, 55.30, 73.96, and 77.18 are consistent with the peaks corresponding to the crystal plane of (220), (311), (222), (400), (422), (620), and (533), respectively [42]. After the anion exchange reaction, the diffraction peaks of the obtained sample corresponding to the cubic Co_3_S_4_ phase (JCPDS card No. 76-1802) can be distinguished in the XRD graph (Figure 1b). Noticeably, the diffraction peaks at 31.24, 37.92, 49.88, 54.94, 75.54, and 78.10 associated with reflections from (311), (400), (511), (440), (642), and (553) planes. This shows that we have successfully synthesized cubic-Co_3_S_4_ from Co_3_O_4_ and are also in agreement with the standard JCPDS (card No. 73-1703) data with space group: Fd3-m (227) [43]. To further unveil the formation of Co_3_S_4_ from Co_3_O_4_, we conducted ex situ Raman experiments for both Co_3_O_4_ and Co_3_S_4_ and their spectra are shown in Figure 1c. Spectra Co_3_O_4_ samples display five characteristic Raman peaks corresponding to the F_2g_^1^ (~194 cm^−1^), E_g_ (~491 cm^−1^), F_2g_^2^ (~525 cm^−1^), F_2g_^3^ (~620 cm^−1^), and A_1g_ (~694 cm^−1^) phonon modes [44]. Following the formation of cubic Co_3_S_4_ from Co_3_O_4_ through the anion exchange reaction, four significantly changed Raman peak signals were observed in the spectra, as shown in Figure 1c. The observed four phonon modes of Co_3_S_4_ were F_2g_^1^ (~154 cm^−1^), E_g_ (~240 cm^−1^), F_2g_^3^ (~340 cm^−1^), and A_1g_ (~389 cm^−1^).

### 3.2. Morphological and Compositional Properties

**Figure 2:**
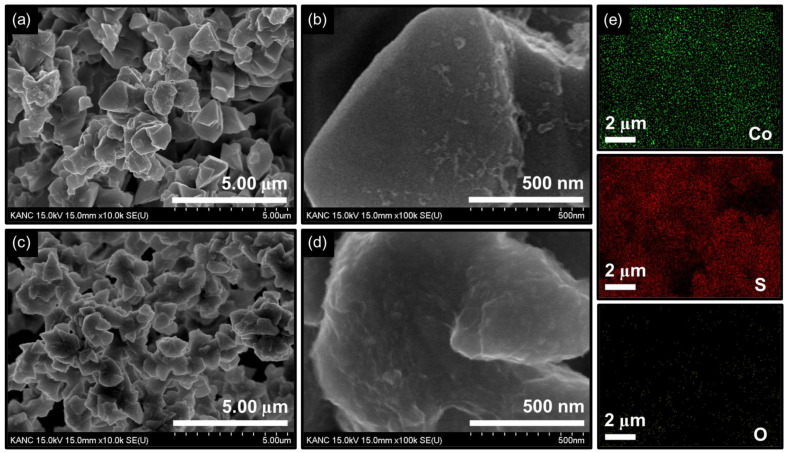
Low and high magnifications FE-SEM images of (**a**,**b**) Co_3_O_4_ and (**c**,**d**) Co_3_S_4_ electrode films. (**e**) FE-SEM-EDX mapping images for the transformed Co_3_S_4_ electrode films.

The morphologies, microstructures, and elemental distribution of the materials at microstructural levels of Co_3_O_4_ and Co_3_S_4_ were analyzed by FE-SEM and FE-SEM-EDX mapping. Figure 2 presents the FE-SEM images of the Co_3_O_4_ and Co_3_S_4_ films. The Co_3_O_4_ electrode film exhibits a polyhedron-like 3D architecture that is randomly stacked and is vertically grown on the pre-cleaned substrate (NF), leading to voids surrounding the irregularly grown polyhedrons during the hydrothermal process, whereas variations of polyhedron size and rougher surface were noted for Co_3_S_4_ films. This change from a smoother surface of Co_3_O_4_ to a much rougher surface for Co_3_S_4_ might be the result of a significant change in the size of polyhedrons during recrystallization during the anion exchange process. Furthermore, SEM-EDX was utilized to analyze the elemental composition and distribution of materials at microstructural levels. Figure S1 shows the obtained EDX spectra of Co_3_O_4_ and Co_3_S_4_ electrode films, and their extracted chemical composition and distribution summed up in the inset of Figure S1, revealing the stoichiometric chemical compositions of the respective electrode film.

### 3.3. Chemical State Characteristics

**Figure 3:**
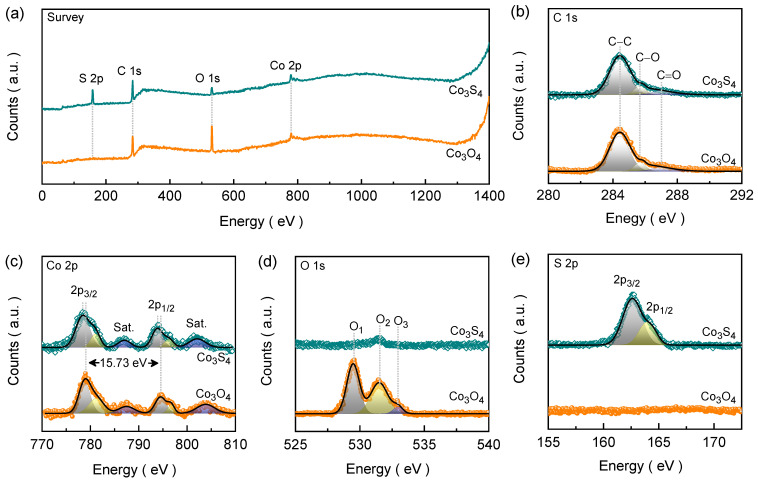
(**a**) High-resolution XPS survey spectra and the narrow-ranged XPS emission spectra for (**b**) C 1s (**c**) Co 2p, (**d**) O 1s, and (**e**) S 2p of the Co_3_O_4_ and Co_3_S_4_ electrode films. The high-resolution emission spectra were deconvoluted using the Gaussian curve fitting model.

For the detailed investigation of surface chemistry, XPS was further employed to analyze the elemental composition, chemical states, and electronic structure of Co_3_O_4_ and Co_3_S_4_ electrode films. All of the obtained core-level XPS emission spectra were best fitted using the Gaussian curve fitting model to reconstruct the XPS spectra. Figure 3a shows the survey spectra of Co_3_O_4_ and Co_3_S_4_ electrode films. The Co_3_O_4_ spectra show three peaks located at 284.30, 531.07, and 779.69 eV, corresponding to the C 1s, O 1s, and Co 2p degenerate states. However, after the phase transformation, an additional emission peak related to S 2p was observed at 162.84 eV. The additional C 1s peak apart from the constituent elements’ emission aroused from the contaminated carbon present inside the vacuum chamber. Figure 3c shows the Co 2p core-level spectra for Co_3_O_4_ and Co_3_S_4_ electrode films, which display a total of four emission peaks where the two most intense peaks of four are deconvoluted into doublets. These six emission peaks of Co_3_O_4_ were located at 778.78, 781.27, 787.46 (satellite [Sat.]), 794.51, 796.90, and 803.83 (Sat.) eV. The higher and lower energy peaks correspond to Co 2p_3_/_2_ and Co 2p_1_/_2_, respectively. The energy separation of 15.73 eV between Co 2p_1_/_2_ and Co 2p_3_/_2_ confirms a mixed Co^3^⁺ and Co^2^⁺ oxidation state present in the Co_3_O_4_ structure [30,45]. Figure 3d shows the O 1s spectrum of Co_3_O_4_, which shows two superimposed peaks deconvoluted into three. The first peak present at 529.47 eV represents lattice oxygen (O_1_), the second peak positioned at 531.49 eV originated from the oxygen vacancies (O_2_), and the third peak located at 532.95 eV corresponds to chemisorbed/dissociated oxygen species (O_3_) [46]. As shown in Figure 3e, following the anion exchange, the formation of the new S 2p signal appears for the Co_3_S_4_ film while the previous O 1s peaks diminish and nearly vanish. Two characteristic peaks were observed at 162.74 (S 2p_3/2_) and 163.93 (S 2p_1/2_) eV, respectively. The 1.19 eV energy difference between S 2p_1_/_2_ and S 2p_3_/_2_ indicates the presence of divalent sulfur (S^2−^) bonded to Co^2^⁺ and Co^3^⁺ in Co_3_S_4_ [47]. The weak O 1s peaks suggest partial surface oxidation, which might arise due to air oxidation on the surface during sample preparation. The separation energy between Co 2p_1_/_2_ and Co 2p_3_/_2_ in Co_3_S_4_ remains almost consistent with that in Co_3_O_4_. Thus, the formation of pure Co_3_O_4_ and its subsequent conversion into Co_3_S_4_ is confirmed using XPS analysis.

### 3.4. Electrocatalytic OER Performances

**Figure 4:**
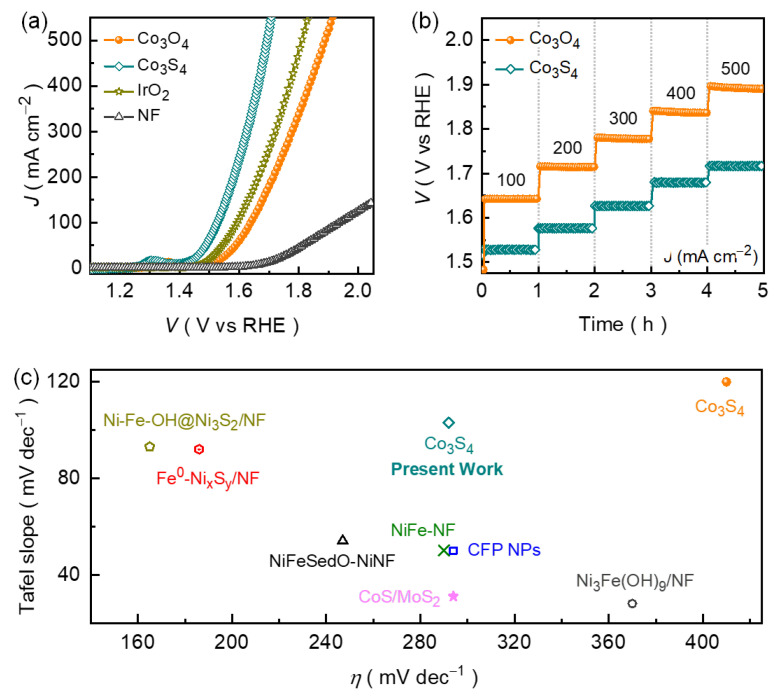
(**a**) LSV curves and (**b**) voltage-step profile for the Co_3_O_4_ and Co_3_S_4_ catalyst films. (**c**) Comparative electrocatalytic OER performance of various cobalt-based catalysts with the proposed polyhedron-like Co_3_S_4_ catalyst measured at a current density of 100 mA cm^−2^.

The catalytic performance of the formed polyhedron-like Co_3_O_4_ and Co_3_S_4_ catalyst films were evaluated using the LSV curves, which reveals the significant variation in the redox properties and the catalytic OER performances after the substitution of sulfide ions in the oxide cocatalyst material. For comparison, the control catalysts including a commercial IrO_2_ benchmark supported on NF substrate as well as bare NF substrate were also examined at the same experimental condition. The selection of reference catalysts provides a thorough basis for evaluating the catalytic OER performance of the proposed materials, with IrO_2_ serving as a high-activity standard and the bare NF offering a baseline to understand the contributions of the constituent components to the overall catalytic behavior. Figure 4a shows the typical *JR* compensated OER LSV curves for the Co_3_O_4_, Co_3_S_4_, NF substrate, and IrO_2_ catalyst films recorded in an alkaline KOH solution (1.0 M) medium. The LSV curve of the bare NF substrate exhibited a very low OER current response throughout the potential sweep compared to the formed catalysts, suggesting that the NF substrate itself was not electrochemically active. Interestingly, the polyhedron-like Co_3_S_4_ catalyst exhibited superior electrocatalytic activity compared to the other catalyst films, achieving a high current density of 100 mA cm^−2^ at a small overpotential of 292 mV (vs. RHE). Evidently, this overpotential value is lower than the pure Co_3_O_4_ (410 mV vs. RHE) catalyst and NF substrate (708 mV vs. RHE), and even surpasses the catalytic activity of the IrO_2_ (362 mV vs. RHE) catalysts at the same current density.

Moreover, the overpotentials required to drive the current densities of 200, 300, 400, and 500 mA cm^−2^ for the polyhedron-like Co_3_S_4_ catalyst were 352, 395, 431, and 463 mV, respectively. However, to drive the same anodic current densities, the required overpotentials for the Co_3_O_4_ catalyst were 483, 546, 604, and 657 mV (vs. RHE). The smaller overpotentials of the Co_3_S_4_ catalyst at all anodic current densities demonstrated its superior catalytic OER activity compared to that of the pure Co_3_O_4_ catalyst. The noticeable improvement in the catalytic activity of the Co_3_S_4_ catalyst suggests that the substitution of sulfur with oxygen constituent enhances the material conductivity (Figure S2) and elevates the accessible active sites (Figure S3), thereby improving electron transfer throughout the polyhedron network [48,49,50]. This increased electron mobility leads to faster reaction kinetics, as the sulfur atoms modify the electronic properties, lowering energy barriers, and facilitating more efficient catalytic reactions [51,52,53].

The overpotentials of the Co_3_O_4_ and Co_3_S_4_ catalysts at various current densities can also be assessed from their respective chronopotentiometric rate performance curves. Figure 4b shows the chronopotentiometric voltage-step profile curve being the function of current density. The applied current density rate was constantly maintained for an hour at 100 mA cm^−2^ and then gradually increased up to 500 mA cm^−2^ with an interval of 100 mA cm^−2^ to highlight the potential response at varied current rates. Both electrocatalysts exhibit static potential response throughout the voltage-step profile and demonstrate the direct relationship between the potential and applied current density. Clearly, the Co_3_S_4_ catalyst achieved a smaller potential response at each voltage step compared to the pure Co_3_O_4_ catalyst, which is a result of efficient mass transport with improved conductivity (Table 1). These results are further supported by the *ECSA*-compensated LSV curve (Figure S4a), which reveals the Co_3_S_4_ catalyst consistently maintains a lower potential response at each driven *ECSA*-compensated current density (*J_ECSA_*), further confirming the superior catalytic OER performance of Co_3_S_4_ than Co_3_S_4_ catalyst. Besides, the Co_3_S_4_ catalyst demonstrated the competitive catalytic OER activity compared to the other noble metal-free catalysts in an alkaline KOH medium (Figure 4c and Table S1).

**Figure 5:**
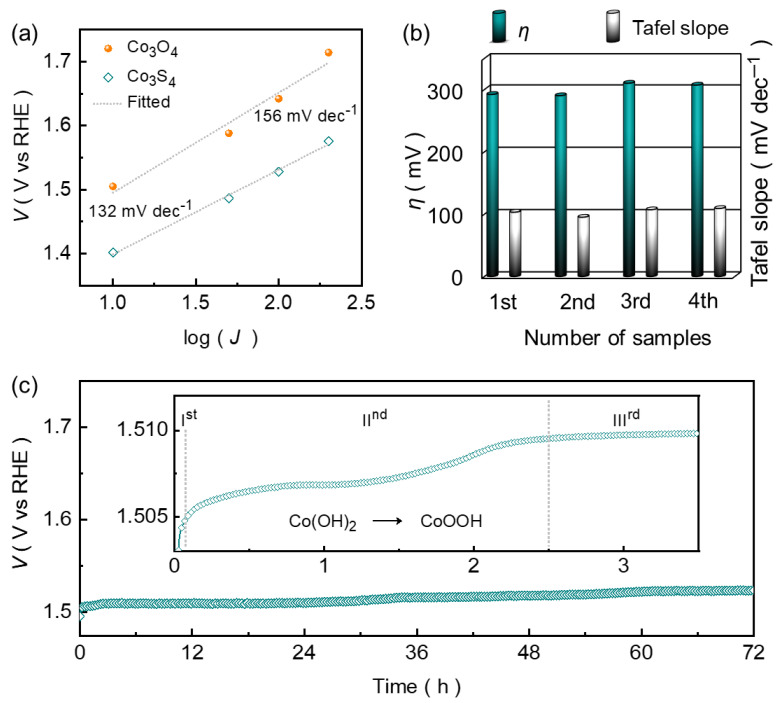
(**a**) Tafel slope for the Co_3_O_4_ catalyst films. (**b**) Reliability of the Co_3_S_4_ for the catalytic OER. (**c**) Prolonged chronopotentiometric OER stability of Co_3_S_4_ catalyst measured up to 72 h at a current density of 100 mA cm^−2^.

The variation in the overpotential is directly associated with the enhanced reaction kinetics, which can be better understood by examining the Tafel curves. The steepness of the Tafel curve provides insight into the reaction rate kinetics and the efficiency of the catalyst. Figure 4b presents the Tafel plots derived from the chronopotentiometric curves using the Tafel equation:

*ɳ* = *α* + [log (*J*) × *β*], (4)

where *α*, *J*, and *β* represent the constant of the equation, current density, and the Tafel slope, respectively. A comparatively small Tafel slope of 132 mV dec^−1^ was estimated for the Co_3_S_4_ catalyst compared to the pure Co_3_O_4_ (156 mV dec^−1^) catalyst, highlighting the superior catalytic efficiency and rapid OER kinetics of the catalyst electrode after the anion exchange process. The distinct reduction in the Tafel slope was aroused due to the enhanced material conductivity and simultaneously increased a larger number of catalytically active sites which are associated with the substitution of sulfur by oxygen, resulting in the enhanced catalytic OER reaction kinetics. Notably, the catalytic OER performances of the Co_3_S_4_ catalyst were found to be highly reliable, as demonstrated in the sequential independent tests (Figure 5b). Moreover, the intrinsic OER reaction kinetics of the catalyst material is also closely related to their respective turnover frequency (TOF), which further quantifies the efficiency of the catalyst by measuring the number of molecules that react at the available electrocatalytically active sites per unit of time and can be calculated using the following equation:

*TOF* = [*J* × *A*]/[*n* × *F* × *N*], (5)

where “*n*” stands for the number of moles of the active electrocatalyst, which can be determined based on the loading weight and molecular weight of the active catalyst material deposited on the NF substrate. The value of *n* is 8.31 and 6.56 × 10^−6^ moles for Co_3_O_4_ and Co_3_S_4_, respectively. The factor “*N* (for OER it is 4)” accounts for the four electrons involved per mole of oxygen in the reaction. “*A*” and “*F*” denote the active catalyst deposition area (cm^2^) and the Faraday constant (96,485.3329 A·s mol^−1^). Figure S3a shows the *TOF* curves for the Co_3_O_4_ and Co_3_S_4_ catalysts obtained from the measured LSV curves. A significantly higher *TOF* of 0.2826 s^−1^ was calculated for the Co_3_S_4_ catalyst at 1.739 V (vs. RHE) compared to the Co_3_O_4_ catalyst (0.0739 s^−1^). This enhancement is approximately four-fold greater than the pristine catalyst at the same driving potential, indicating that the sulfur substitution effectively enhances the reaction kinetics by promoting more efficient electron and ion transport throughout the catalyst network.

Apart from the catalytic ascendency of the Co_3_S_4_ for OER, the long-term durability in alkaline medium is the characteristic feature of an efficient catalyst for their practical utilization. Figure 5c shows the prolonged chronopotentiometric stability performance of the polyhedron-like Co_3_S_4_ catalyst toward the OER in an alkaline KOH medium. The chronopotentiometric stability curve was recorded at an applied current density of 100 mA cm⁻^2^ with continuous electrolysis for up to 72 h. The Co_3_S_4_ catalyst exhibited quite a stable voltage response during the vigorous and continuous gas bubble evolution at a high current density of 100 mA cm^−2^, which is attributed to efficient electron and ion transport demonstrating the strong durability during the prolonged catalytic OER test. This analysis is further supported by the EIS curve (Figure S4c), which shows the insignificant change in charge transfer resistance after the prolonged catalytic OER testing. Moreover, an almost identical LSV curve (Figure S4d) further confirmed its exceptional long-term OER performance in an alkaline environment.

Conspicuously, the chronopotentiometric curve reveals the small potential loss at the beginning of the test, which is shown in the inset of Figure 5c. This dramatic change in the potential is associated with the in situ partial phase transformation of an active Co_3_S_4_ catalyst into CoOOH at the surface upon electrooxidation process forming the CoOOH@Co_3_S_4_ heterostructure, which serves as the active catalytic center and is typical for Co-based catalyst during the OER process [54]. This phase transformation can be quantitively understood from the three distinct potential regions (inset of Figure 5c). To gain further insight into the phase transformation during the catalytic OER process, the ex-situ EDX (Figure S5a), Raman (Figure S5b), and the narrow XPS (Figure S6) spectra were recorded after the prolonged chronopotentiometric stability to understand the changes in the chemical composition and chemical bonds of the catalyst. The EDX spectrum revealed the sulfur deficiency in the constituent composition with an abrupt rise in the oxygen percentage, which is obvious during the OER in an alkaline medium. This analysis is consistent with the post-stability measured XPS emission spectra, which further revealed the decreased S 2p peak (Figure S6b) intensity caused due to the depletion of sulfur and the simultaneously increased O 1s peak (Figure S6c) intensity upon electrooxidation process in an alkaline KOH medium [40]. Besides, the spin-energy separation of Co 2p_3_/_2_ and Co 2p_1_/_2_ (Figure S6a) degenerate state reduced to 15.31 eV, indicating the partial alteration of Co^2+^ into Co^3+^ during the chronopotentiometric stability, as illustrated in the inset of Figure 5c. Further, the Raman spectrum reveals an additional peak positioned at ~505 cm^−1^, originated due to the presence of CoOOH species, validating the catalyst phase transformation during the catalytic OER process.

## 4. Conclusions

The polyhedron-like Co_3_S_4_ phase was transformed successfully via an anion exchange process of the Co_3_O_4_ template using Na_2_S, which was synthesized through a cost-effective and eco-friendly hydrothermal process followed by calcination in ambient air. The polyhedral Co_3_S_4_ structure served as the highly efficient OER catalyst in an alkaline electrolyte environment. A comprehensive electrocatalytic OER study concludes that the synergistic contribution from the multivalence cobalt state, 3D polyhedral structure, and improved material conductivity support plays a crucial role in enhancing catalytic kinetics, primarily by providing better active site accessibility, *TOF*, and improved electron transport. The optimized Co_3_S_4_ catalyst demonstrated excellent catalytic OER activity in alkaline 1.0 M KOH condition by achieving a significantly reduced overpotential of 292 mV at a high current density of 100 mA cm^−2^ with the small Tafel slope of 132 mV dec^−1^ compared to the pure Co_3_O_4_ (410 mV and 156 mV dec^−1^) catalyst along with the higher normalized *J_ECSA_* values at each potential. Further, the Co_3_S_4_ catalyst maintains the static potential response between the current density rate of 100 and 500 mA cm^−2^ and reveals excellent prolonged endurance up to 72 h at 100 mA cm^−2^. A notable phase transformation of an active Co_3_S_4_ catalyst into CoOOH@Co_3_S_4_ was observed after the chronopotentiometric stability test, which was affirmed through the post-stability measured EDX, Raman, and XPS spectra.

## Figures and Tables

**Table 1:** The EIS curves fitted parameter values obtained using Z-view software (serial #13339, Scribner Associates, Inc., Southern Pines, NC, USA) for the pure Co_3_O_4_ and Co_3_S_4_ catalysts (*Rct*; change in charge transfer resistance).

Electrocatalyst	Before Catalytic OER Stability		After Catalytic OER Stability	
	*Rs* (Ω)	*Rct* (Ω)	*Rs* (Ω)	*Rct* (Ω)
Co_3_O_4_	0.435	7.29	-	-
Co_3_S_4_	0.386	4.48	0.401	4.72

## Data Availability

The data presented in this study are available on reasonable request from the corresponding author.

## Supplementary Materials

The following supporting information can be downloaded at: https://www.mdpi.com/2079-4991/14/21/1732/s1, Figure S1: EDX spectra; Figure S2: EIS curves; Figure S3: Non-Faradaic CV curves for the estimation of *ECSA*; Figure S4: *ECSA*-compensated LSV curves, TOF curves, and post-stability recorded LSV and EIS curves; Figure S5: post-stability recorded EDX and Raman spectra; Figure S6: post-stability recorded XPS spectra. Table S1: The electrocatalytic OER performance of our optimized Co_3_S_4_ catalyst films and other metal sulfide-based catalyst in alkaline electrolyte medium at 100 mA cm^−2^ [55,56,57,58,59,60,61,62].
