# Correction: Ahmed et al. In Situ Transformed CoOOH@Co_3_S_4_ Heterostructured Catalyst for Highly Efficient Catalytic OER Application. *Nanomaterials* 2024, *14*, 1732

**DOI:** 10.3390/nano15221705

**Published:** 2025-11-12

**Authors:** Abu Talha Aqueel Ahmed, Vijaya Gopalan Sree, Abhishek Meena, Akbar I. Inamdar, Hyunsik Im, Sangeun Cho

**Affiliations:** 1 Division of System Semiconductor, Dongguk University, Seoul 04620, Republic of Korea; abutalha.aa@dongguk.edu (A.T.A.A.); akbarphysics@dongguk.edu (A.I.I.); hyunsik7@dongguk.edu (H.I.); 2 Department of Physics, Dongguk University, Seoul 04620, Republic of Korea; sreevg@dongguk.edu

In the original publication [1], reference 5, “Hassan, Q.; Algburi, S.; Sameen, A.Z.; Salman, H.M.; Jaszczur, M. Green hydrogen: A pathway to a sustainable energy future. *Int. J. Hydrogen Energy* **2024**, *50*, 310–333”, was retracted prior to this publication, and the authors would like to replace it with a new one:

Hassan, N.S.; Jalil, A.A.; Rajendran, S.; Khusnun, N.F.; Bahari, M.B.; Johari, A.; Kamaruddin, M.J.; Ismail, M. Recent review and evaluation of green hydrogen production via water electrolysis for a sustainable and clean energy society. *Int. J. Hydrogen Energy* **2024**, *52*, 420–441. https://doi.org/10.1016/j.ijhydene.2023.09.068.

With this correction, the order of the references has not been adjusted. The authors state that the scientific conclusions are unaffected. This correction was approved by the Academic Editor. The original publication has also been updated.
