# Supplementary material for: In Situ Transformed CoOOH@Co3S4 Heterostructured Catalyst for Highly Efficient Catalytic OER Application
Source: Nanomaterials (Basel). 2024 Oct 29;14(21):1732. doi: 10.3390/nano14211732 (PMC11547189; doi:10.3390/nano14211732)
Supplement: Supplementary file 1 [file nanomaterials-14-01732-s001.zip › nanomaterials-3263753-supplementary.pdf]

Supporting Information

In Situ Transformed $CoOOH@Co_3S_4$ Heterostructured Catalyst for Highly Efficient Catalytic OER Application

Abu Talha Aqueel Ahmed [1], Vijaya Gopalan Sree [2], Abhishek Meena [1], Akbar I. Inamdar [1], Hyunsik Im [1] and Sangeun Cho [1*]

[1] *Division of System Semiconductor, Dongguk University, Seoul 04620, Republic of Korea*
[2] *Department of Physics, Dongguk University, Seoul 04620, Republic of Korea*

Corresponding Author: sangeun.c@dongguk.edu

Table S1. The electrocatalytic OER performance of our optimized Co_3S_4 catalyst films and other metal sulfide–based catalyst in alkaline electrolyte medium at 100 mA cm^{-2}.

No.	Catalyst film	Overpotential @100 (mA cm^{-2})	Tafel slope (mV dec $^{-1}$)	Stability at J (J in mA cm^{-2})	Supporting Reference
1	Ni-Fe-OH@Ni_3S_2/NF	165 mV	93	50 h@100	[55]
2	$Ni_{50}Fe_{50}$-DAT	300 mV	–	72 h@100	[56]
3	NiFeSedO-NiNF	247 mV	54	~8 h@100	[57]
4	Fe^0-Ni_xS_y/NF	186 mV	92	10 h@1.47 V	[58]
5	CFP NPs	294 mV	50	70 h@100	[59]
6	$Ni_3Fe(OH)_9$/NF	370 mV	28	10 h@100	[60]
7	CoS/MoS_2 heterostructure	294 mV	31	100 h@100	[61]
8	NiFe-NF	290 mV	50.1	30 h@100	[62]
9	Polyhedron-like Co_3S_4	292 mV	103	101	Present Work

Supporting Figures

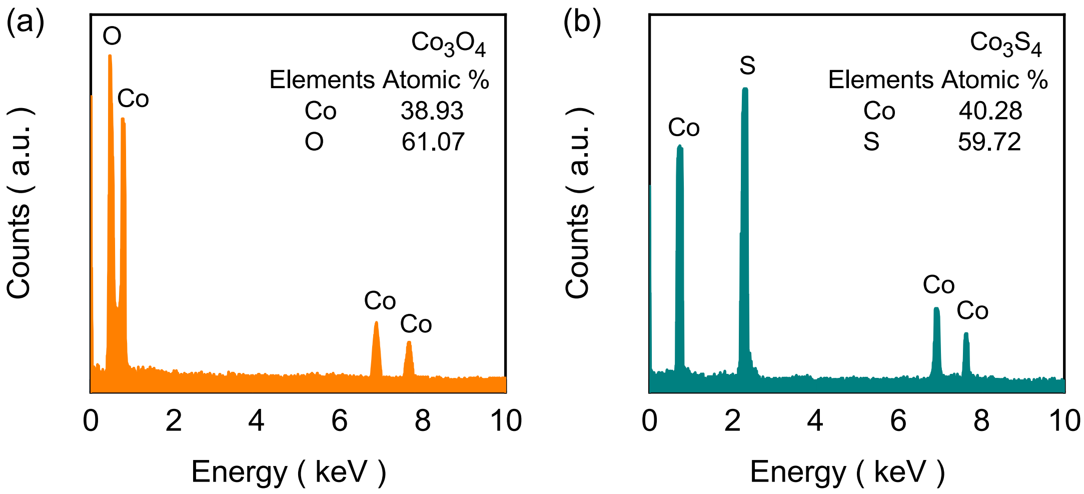

Figure S1. (**a**) EDX spectra of (a) Co_3O_4 and (**b**) Co_3S_4 electrode films. Notably, the inset table shows the obtained elemental compositions in the atomic percentage ratios.

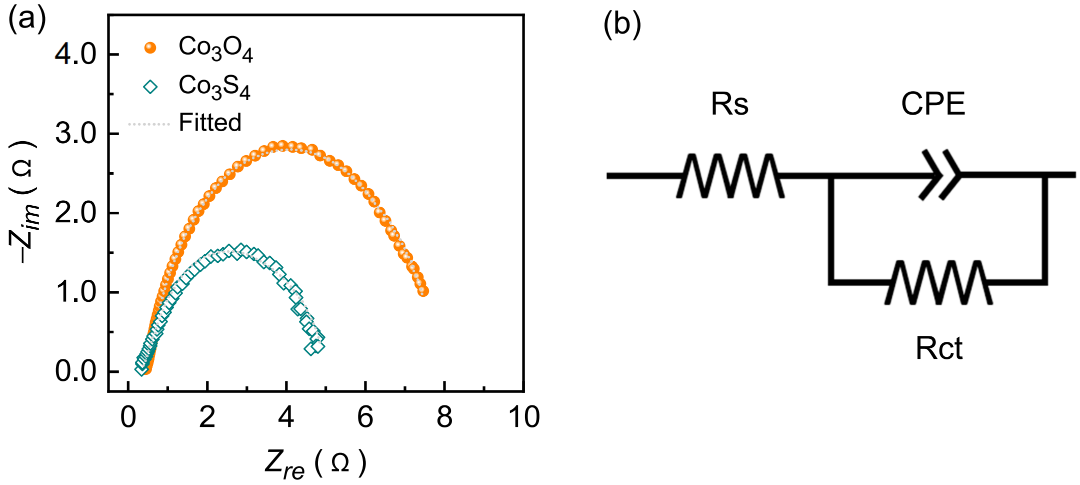

Figure S2. (**a**) EIS curves recorded for the Co_3O_4 and Co_3S_4 electrode films along with the fitting curves date marked with the grey dotted lines. (**b**) The formed tank-circuit used to fit the EIS curves in Z-view software.

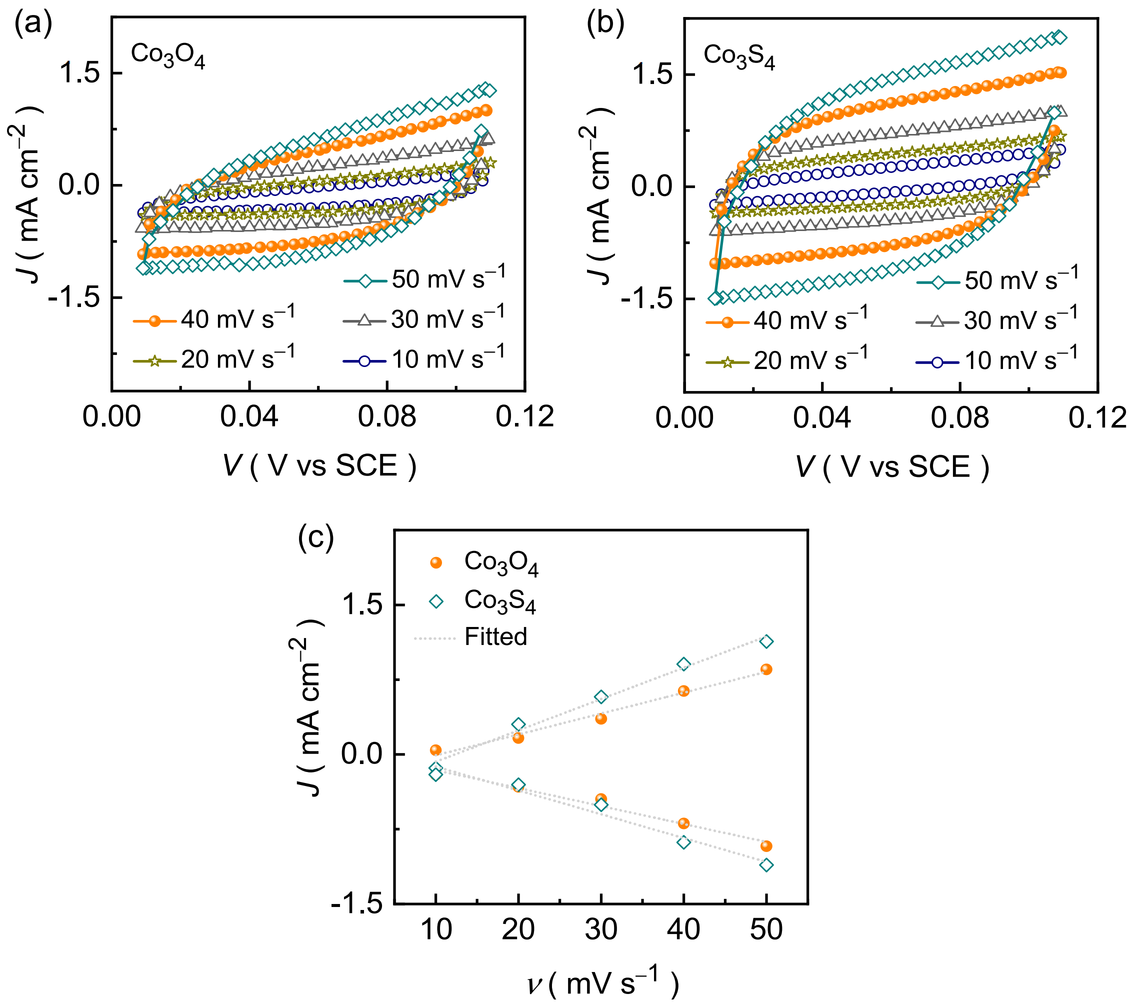

Figure S3. Scan rate dependent CV curves of the (a) Co_3O_4 and (b) Co_3S_4 catalyst films measured in non-Faradaic potential region at different scan rates. (c) "*J* versus *v*" plots obtained at 0.06 V (*vs*. SCE) from non-Faradaic CV curves to calculate the double-layer capacitance and *ECSA*.

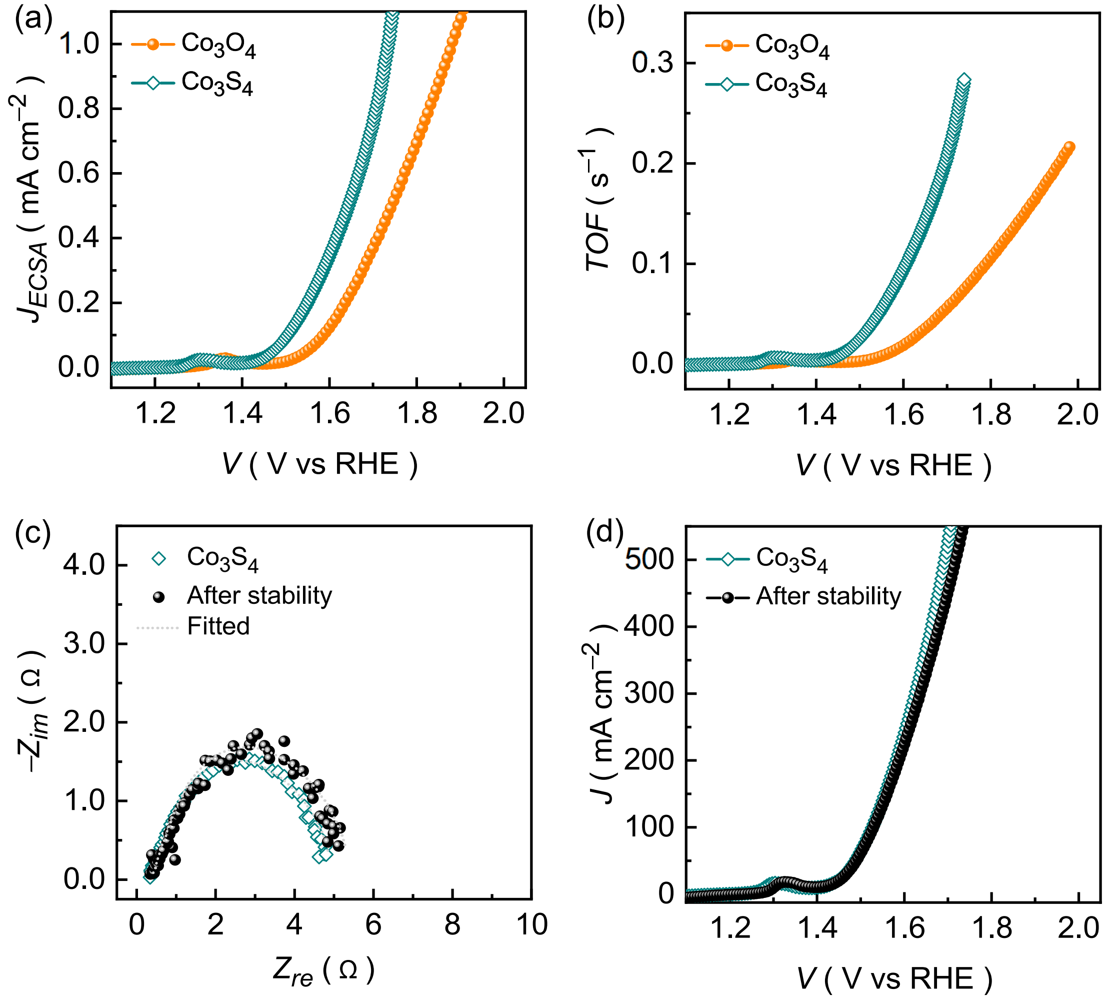

Figure S4. (a) *ECSA*-compensated LSV curves and (**b**) *TOF* curves of Co_3O_4 and Co_3S_4 catalyst films. Post-stability measured (**c**) EIS and (**d**) LSV curves of the proposed Co_3S_4 catalyst film.

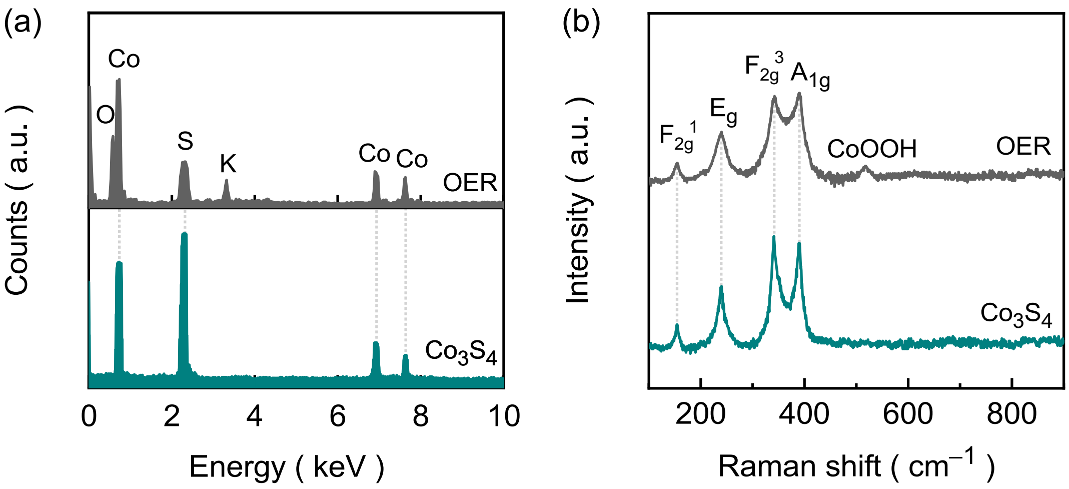

Figure S5. (**a**) EDX and (**b**) Raman spectra of Co_3S_4 catalyst film measured after the prolonged chronopotentiometric stability test.

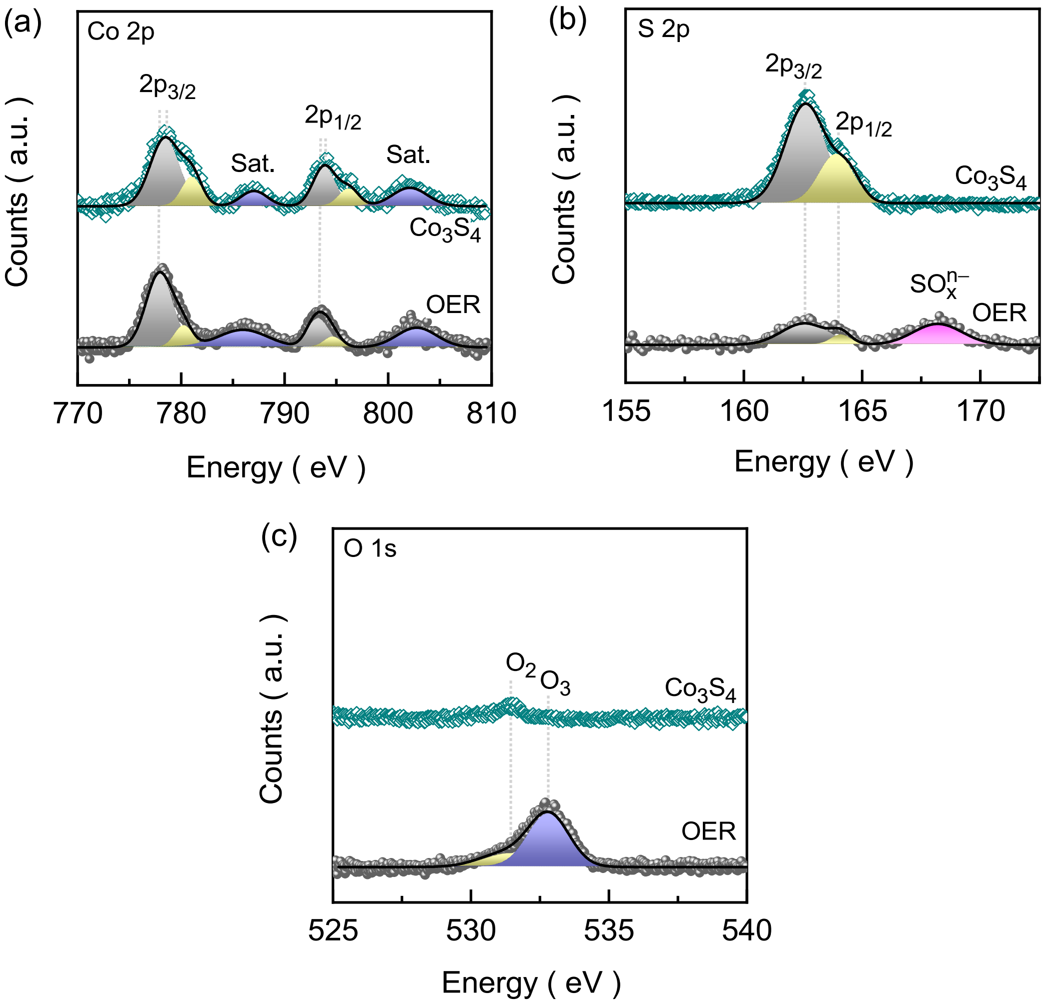

Figure S6. High-resolution (**a**) Co 2p, (**b**) S 2p, and (**c**) O 1s XPS emission spectra of Co_3S_4 catalyst film measured after the prolonged chronopotentiometric stability test.

Supporting References

55. Zou, X.; Liu, Y.; Li, G.-D.; Wu, Y.; Liu, D.-P.; Li, W.; Li, H.-W.; Wang, D.; Zhang, Y.; Zou, X. Ultrafast Formation of Amorphous Bimetallic Hydroxide Films on 3D Conductive Sulfide Nanoarrays for Large-Current-Density Oxygen Evolution Electrocatalysis. *Adv. Mater.* **2017**, *29*, 1700404.
56. Hoang, T.T.H.; Gewirth, A.A. High Activity Oxygen Evolution Reaction Catalysts from Additive-Controlled Electrodeposited Ni and NiFe Films. *ACS Catal.* **2016**, *6*, 1159–1164.
57. Peugeot, A.; Creissen, C.E.; Karapinar, D.; Tran, H.N.; Schreiber, M.; Fontecave, M. Benchmarking of oxygen evolution catalysts on porous nickel supports. *Joule* **2021**, *5*, 1281–1300.
58. Cheng, X.; Lei, C.; Yang, J.; Yang, B.; Li, Z.; Lu, J.; Zhang, X.; Lei, L.; Hou, Y.; Ostrikov, K. Efficient Electrocatalytic Oxygen Evolution at Extremely High Current Density over 3D Ultrasmall Zero-Valent Iron-Coupled Nickel Sulfide Nanosheets. *ChemElectroChem* **2018**, *5*, 3866–3872.
59. Meena, A.; Aqueel, A.A.T.; Narayan, S.A.; Gopalan, S.V.; Im, H.; Cho, S. Highly Efficient CoFeP Nanoparticle Catalysts for Superior Oxygen Evolution Reaction Performance. *Nanomaterials* **2024**, *14*, 1384.
60. Lu, X.; Zhao, C. Electrodeposition of hierarchically structured three-dimensional nickel–iron electrodes for efficient oxygen evolution at high current densities. *Nat. Commun.* **2015**, *6*, 6616.
61. Ahmed, A.T.A.; Lee, C.H.; Ansari, A.S.; Pawar, S.M.; Han, J.; Park, S.; Shin, G.; Yeon, S.; Cho, S.; Seol, J.; et al. Hybridized heterostructure of CoS and MoS_2 nanoparticles for highly-efficient and robust bifunctional water electrolysis. *Appl. Surf. Sci.* **2022**, *592*, 153196.
62. Guo, D.; Qi, J.; Zhang, W.; Cao, R. Surface Electrochemical Modification of a Nickel Substrate to Prepare a NiFe-based Electrode for Water Oxidation. *ChemSusChem* **2017**, *10*, 394–400.
